# PARA: A New Platform for the Rapid Assembly of gRNA Arrays for Multiplexed CRISPR Technologies

**DOI:** 10.3390/cells11162467

**Published:** 2022-08-09

**Authors:** Guoliang Yuan, Stanton Martin, Md Mahmudul Hassan, Gerald A. Tuskan, Xiaohan Yang

**Affiliations:** 1 Biosciences Division, Oak Ridge National Laboratory, Oak Ridge, TN 37831, USA; 2 The Center for Bioenergy Innovation, Oak Ridge National Laboratory, Oak Ridge, TN 37831, USA; 3 Department of Genetics and Plant Breeding, Patuakhali Science and Technology University, Dumki, Patuakhali 8602, Bangladesh

**Keywords:** gRNA array, multiplexed CRISPR, genome editing, assembly method, Golden Gate assembly, PARA, web tool

## Abstract

Multiplexed CRISPR technologies have great potential for pathway engineering and genome editing. However, their applications are constrained by complex, laborious and time-consuming cloning steps. In this research, we developed a novel method, PARA, which allows for the one-step assembly of multiple guide RNAs (gRNAs) into a CRISPR vector with up to 18 gRNAs. Here, we demonstrate that PARA is capable of the efficient assembly of transfer RNA/Csy4/ribozyme-based gRNA arrays. To aid in this process and to streamline vector construction, we developed a user-friendly PARAweb tool for designing PCR primers and component DNA parts and simulating assembled gRNA arrays and vector sequences.

## 1. Introduction

Multiplexed CRISPR technologies are highly effective DNA editing platforms for multigene editing. The three distinct strategies for multiplexed guide RNA (gRNA) expression are: (1) conventional arrayed multiple, individual single gRNA (sgRNA) expression cassettes, in which each sgRNA is transcribed from a separate RNA polymerase III (Pol III) promoter; (2) CRISPR arrays, in which each gRNA is processed via a native CRISPR processing mechanism; and (3) synthetic gRNA arrays, wherein a single RNA transcript is processed post-transcriptionally into multiple individual gRNAs by RNA-cleaving enzymes [1,2]. Notably, synthetic gRNA arrays in genome editing have resulted in higher efficacy of gene disruption in yeast [3], *Drosophila* [4] and plants [5]. Still, a challenge constraining the use of multiplexed CRISPR is the complicated vector design and construction. Although different approaches have been reported for optimizing multiplex gRNA cloning, a series of intermediate vectors and multistep modular cloning are usually required. In fact, creating such arrayed architectures remains technically challenging—partially because of the presence of highly repetitive DNA sequences, which prevent multiplexed CRISPR from being widely adopted in various applications [1]. To address these limitations, we developed the prime assembly of gRNA arrays (PARA) method for the fast cloning of multiple gRNAs in an array into a CRISPR vector via a one-pot reaction in a microcentrifuge tube.

## 2. Materials and Methods

### 2.1. PCR-Based Cloning

The component fragments were PCR-amplified using Q5 High-Fidelity 2X Master Mix (New England Biolabs, Ipswich, MA, USA) with a 65 °C annealing temperature.

### 2.2. Colony PCR

Colony PCR was performed using GoTaq Master Mixes (Promega, Madison, WI, USA) with a 55 °C annealing temperature.

### 2.3. Restriction Digest of Plasmid DNA

The destination plasmid DNA was digested using BsaI-HFv2 (New England Biolabs, Ipswich, MA, USA). This step is useful to increase ligation efficiency for gRNA array with more than two gRNAs.

### 2.4. Gel Purification

The PCR products and digested destination vectors were purified using the Zymoclean Gel DNA Recovery Kit (ZYMO RESEARCH, Irvine, CA, USA).

### 2.5. Golden Gate Assembly

Assembly reactions were performed in a thermocycler using the NEBridge Golden Gate Assembly Kit (BsaI-HFv2) (New England Biolabs, Ipswich, MA, USA) with the suggested assembly protocol.

### 2.6. Plasmid Sequencing

The plasmids were Sanger-sequenced using SimpleSeq Kit Premixed (Eurofins Genomics, Louisville, KY, USA). The sequencing data were aligned with a plasmid sequence in SnapGene.

### 2.7. E. coli Transformation

The *E. coli* transformation was performed using NEB 5-alpha Competent *E. coli* (New England Biolabs, Ipswich, MA, USA), following the manual.

### 2.8. Plasmid Isolation

The plasmid DNA extraction was performed using GenElute Plasmid Miniprep Kit (Sigma-Aldrich, Saint Louis, MO, USA).

### 2.9. Oligos Annealing

The two oligo strands were added together in equal molar amounts. The mixed oligonucleotides were heated to 94 °C for 2 min, then gradually cooled.

### 2.10. Vector Cloning

The U6 promoter in the pKSE401 vector was replaced by a U3 promoter via NEBuilder HiFi DNA Assembly (New England Biolabs, Ipswich, MA, USA), and a window sequence (GGTCGGAGACCAACGGTCTCGGTGGCACCGAGTCGGTGCTTTTTTT) was inserted between the U3 promoter and its terminator. The template vectors were generated by inserting two gBlocks Gene Fragments (IDT, Coralville, IA, USA) into a modified pKSE401 vector via NEBuilder HiFi DNA Assembly. Information for all primers and gBlocks used in this study is provided in Table S1.

### 2.11. Web Tool Design

**Figure 1:**
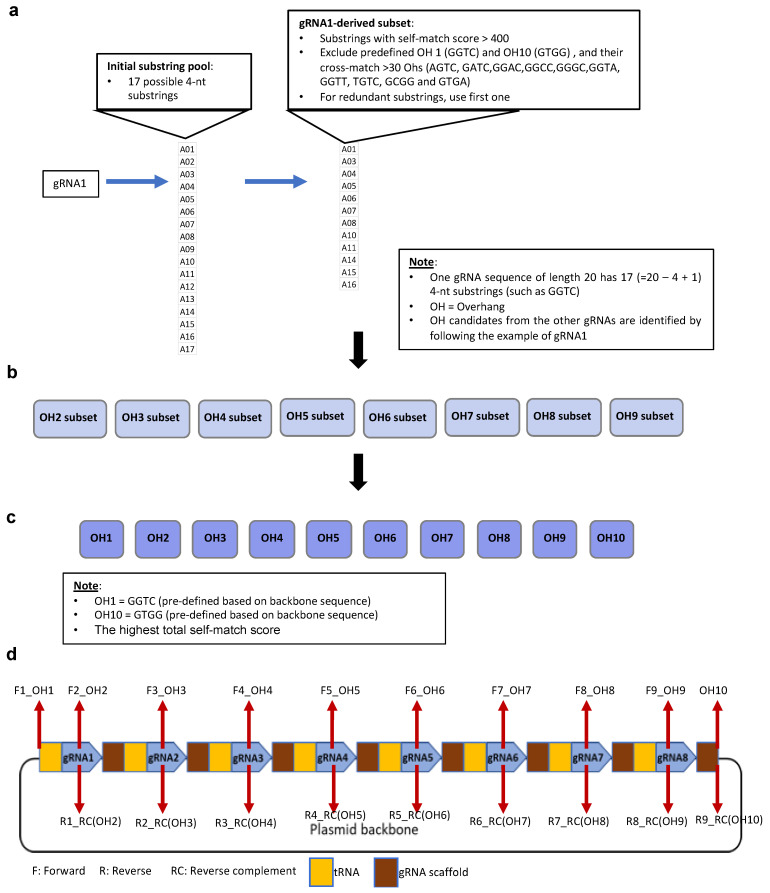
Global optimization of OHs from the 8 gRNA sequences. (**a**) Identification of candidate OHs from each of the 20-nt gRNA sequences. (**b**) Identification of all OH combinations with a pairwise crossmatch score <30 from identified candidate OHs in panel a. (**c**) Identification of the best OH combination with the highest total self-match score for assembling the gRNA array. (**d**) The location of each OH in the gRNA array.

**Figure 2:**
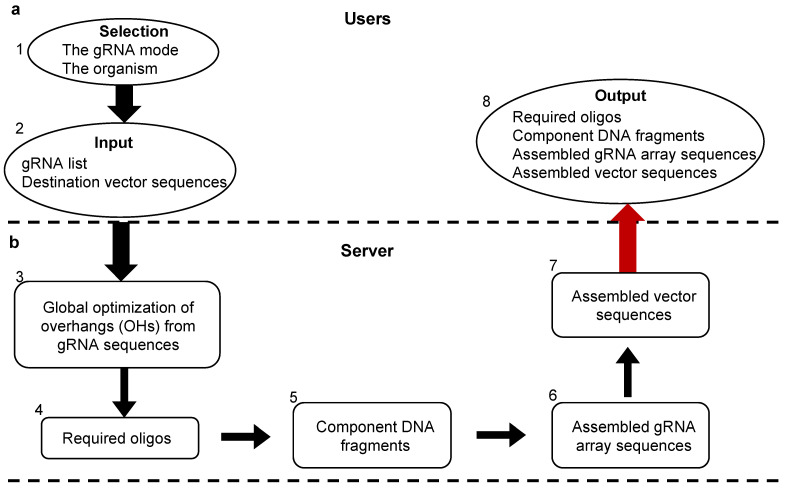
The workflow of PARAweb design. (**a**) The steps on the user front-end side. (**b**) The steps on the server side.

PARAweb is a web tool that provides a complete workflow for the design and assembly of gRNA arrays for multiplex genome editing. PARAweb features a series of drop-down menus that the user may interact with to choose the parameters for the design tool. Parameters include the type of multi-gRNA expression system, the ligation action, the appropriate restriction enzyme and the organism type. After selecting parameters, the user uploads a file containing the gRNA sequences of the gRNA array. The OHs are chosen via algorithm (Figure 1), and a list of primers is displayed in tabular, color-coded format for the PCR amplification of DNA fragments. When the complete sequences are downloaded, DNA constants relevant to specific gRNA mode sets are used. The resulting text files contain the primers, the component DNA fragments of the gRNA array and the complete gRNA array assembly sequence. Figure 2 illustrates the workflow implemented in PARAweb.

For steps 1 and 2, we created the interface of PARAweb, including the name, featured figure, drop-down menus and upload zone. When the defined gRNA sequences and destination vector sequences are given in step 1 and 2, to select high-fidelity OH sets, step 3 is performed for global optimization of OHs from gRNA sequences via: (a) identification of candidate OHs from each of the 20-nt gRNA sequences; (b) identification of all OH combinations with a pairwise crossmatch score <30 from identified candidate OHs in step (a); and (c) identification of the best OH combination with the highest total self-match score for assembling the gRNA array, as illustrated in Figure 1. The crossmatch score and self-match score were used based on the comprehensive profiling of 4 base OH ligation fidelity by T4 DNA ligase [6,7].

Once the OH is selected for each gRNA sequence, the required oligos/primers are generated in step 4. For each primer, the 5′ end of a template-specific sequence is flanked in an orderly manner by 1 BsaI restriction site, 1 specific 4-bp OH sequence and 1 gRNA sequence. In step 5, each component DNA fragment is generated by combining the corresponding forward primer (F [n]), predefined template sequence, and reverse primer (R [n]). In step 6, the assembled gRNA array sequence is generated by combining individual component DNA fragments from step 5. In step 7, assembled vector sequences are generated by connecting the user-provided destination vector and assembled gRNA array sequence from step 6. In step 8, all the described outputs, including the required oligos (step 4), component DNA fragments (step 5), assembled gRNA array sequence (step 6) and assembled vector sequences (step 7), can be downloaded as individual text files.

## 3. Results

**Figure 3:**
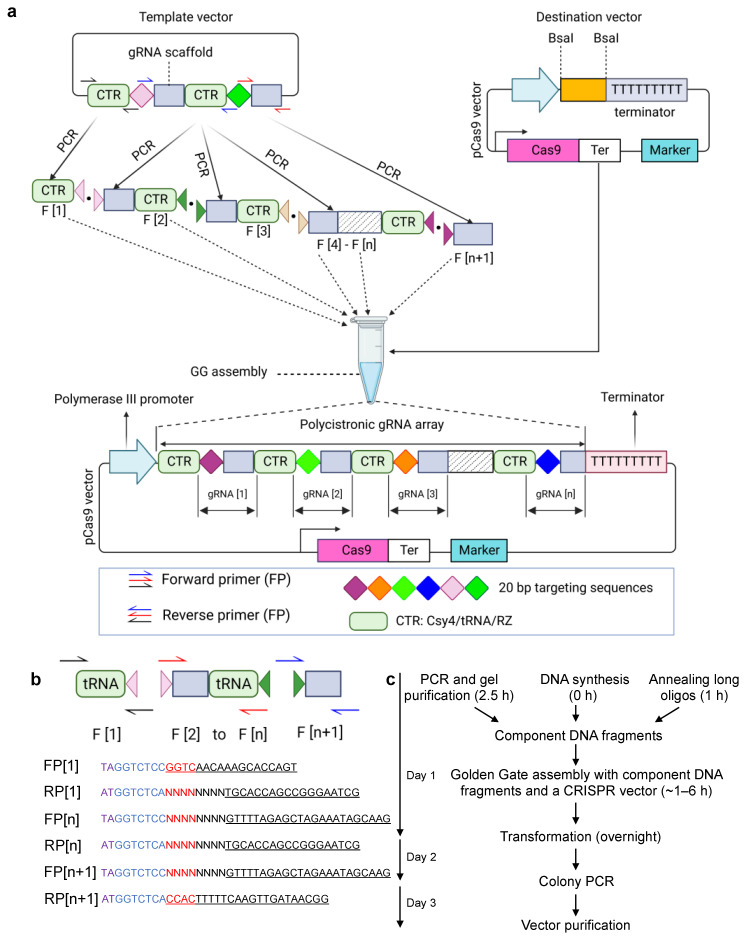
A one-step cloning system for multiplex gRNA expression. (**a**) Golden Gate assembly for preparing a plant binary vector expressing multiple gRNAs under a Pol III promoter. Each tRNA-gRNA unit is amplified from a predesigned template vector. All tRNA-gRNA parts are ligated into a plant binary vector through Golden Gate assembly. (**b**) Design of primers used for amplifying gRNA-tRNA parts. [n + 1] fragments are required for assembly of [n] sgRNAs. Purple sequences are added to enhance the BsaI digestion of PCR products. Blue sequences indicate the BsaI sites. NNNNNNNN sequences are gRNA spacers with lengths ranging from 4 to 20 bp. The red sequences indicate the distinct 4-bp OHs that are required for the ligation of two DNA parts after digestion with BsaI during Golden Gate assembly. Underlined sequences are specific for the template sequence. (**c**) Cloning procedures of multiplex gRNAs.

Generally, directly synthesizing gRNA arrays is challenging because of their highly repetitive elements. Inspired by the multiplexed genome editing with the endogenous transfer RNA (tRNA)–processing system in rice [8], we developed the PCR-based PARA method for the assembly of tRNA-gRNA arrays using Golden Gate (GG) assembly (Figure 3a). To assemble, in an orderly manner, multiple fragments simultaneously, the fragment-specific sequences of 4-base overhangs (OHs) are an essential prerequisite. Unlike the modular cloning with predefined OHs, in the PARA method, the 4-bp OHs are selected from distinct gRNA sequences. Therefore, no scar sequences are introduced during cloning. Thus, the gRNA arrays can be divided into multiple individual DNA parts. Each of the DNA parts can be generated through PCR amplification of a predesigned template vector (Figure 3a). Furthermore, [n + 1] fragments can be used for the assembly of [n] gRNAs. Next, the DNA fragments are ligated, in an orderly manner, into a destination vector containing two predesigned BsaI restriction sites to form a gRNA array within an expression vector (Figure 3a).

One critical step in the PARA method is the design of required oligos (i.e., primers) for the PCR amplification of component fragments. For each primer, the 5′ end of a template-specific sequence is flanked, in an orderly manner, by 1 BsaI restriction site, 1 specific 4-bp OH sequence and 1 gRNA sequence (Figure 3b). Two OHs in the first forward primer and last reverse primer must be complementary with the sticky end of the destination vector digested by BsaI. Moreover, all selected OHs must be distinctive, with low similarity to one another to ensure the orderly assembly of gRNA arrays.

Using the PARA method, the expression vector containing a gRNA array can be constructed within three days, which is, to date, the fastest method for the assembly of gRNA arrays (Figure 3c), saving up to 70% of time and effort in comparison with traditional methods [9,10,11,12]. Depending on the user preference and project requirements, the component DNA fragments can also be generated using commercial DNA synthesis or via annealing long oligos, allowing for high-throughput library synthesis.

**Figure 4:**
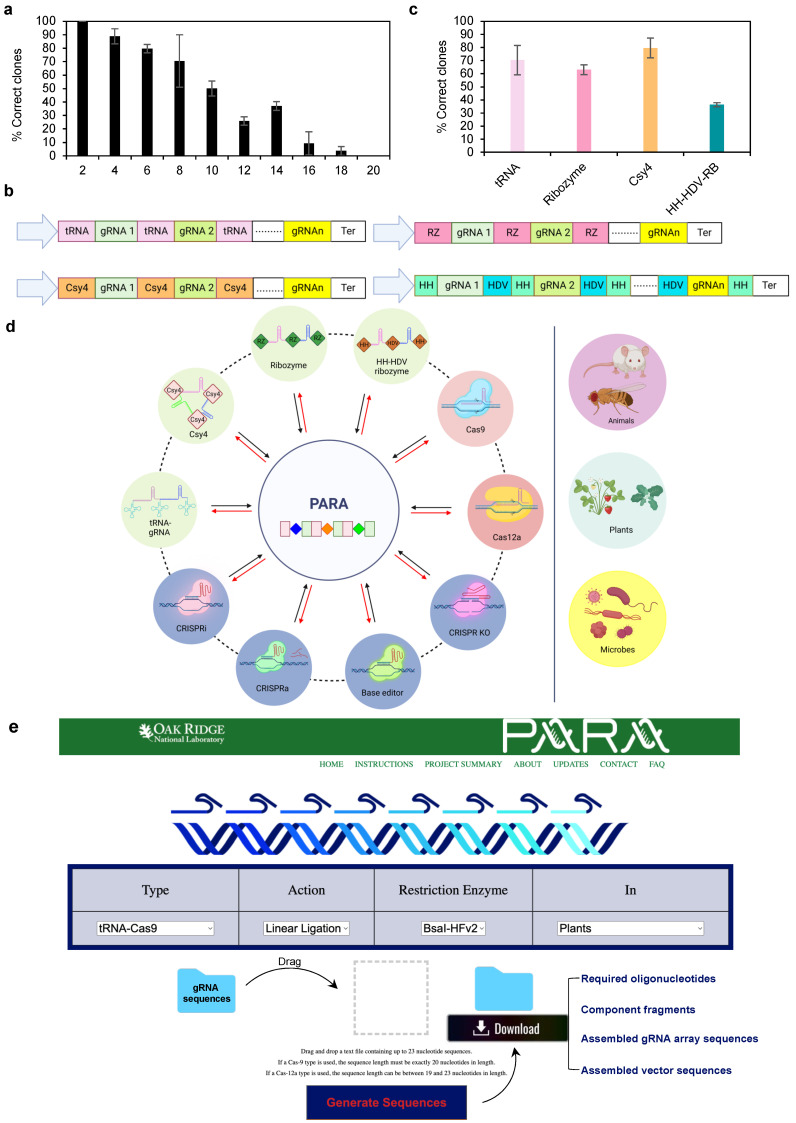
Rapid and highly efficient assembly of the gRNA array. (**a**) Cloning efficiencies of plant tRNA-gRNA systems with different numbers of gRNAs using the PARA method. Error bars represent standard deviations of three replicates. (**b**) Structure of four widely used constructs for expressing gRNA arrays in CRISPR-Cas multiplexed genome editing; tRNA, pre-tRNA^Gly^ gene varying in different organisms; Csy4, 20-bp Csy4 hairpin; RZ, 15-bp RB cleavage site; HH-HDV-RB, HH hammerhead ribozyme and HDV hepatitis delta virus ribozyme. (**c**) The cloning efficiencies of four different gRNA array systems harboring eight gRNAs using the PARA method. Error bars represent the standard deviations of three replicates. (**d**) Features of the PARAweb tool designed for the assembly of gRNA arrays. (**e**) Screenshot of the interface of PARAweb.

To explore the capacity of the PARA method, we performed multi-gRNA assembly with various numbers of gRNAs using the plant tRNA-gRNA system. Four target genes of *Populus deltoides* WV94 were selected from Phytozome [13], and five gRNAs were designed for each gene using a gRNA design web tool, CHOPCHOP [14]. Required oligonucleotides were designed manually as illustrated in Figure 3b. The component fragments were generated through PCR amplification of the predesigned template vector type I followed by gel purification (Figures S1a and S2; Table S2). Then, all component fragments were assembled into a modified pKSE401 vector [15], followed by transformation on day 1. Numerous colonies were observed on the selection medium on day 2 (Figure S3a). Next, we analyzed the colonies via colony PCR (Figure S4) and Sanger sequencing. As expected, the efficiency of GG assembly gradually decreased with the increase in the total number of gRNAs (Figure 4a). In 2-gRNA assembly, target bands were observed in all selected colonies (*n* = 18) (Figure S4a). When the number of gRNAs exceeded 2, false positive colonies were detected on the selection medium (Figure S4b–i). Interestingly, in 4-gRNA assembly, 90% of the transformants harbored correctly assembled constructs (Figure 4a). In the assembly with between 6 and 10 gRNAs, the positive rate of transformants ranged from ~50% to ~80%. To explore the potential of the PARA method, we further studied the assembly of gRNA arrays with up to 20 gRNAs. More than 25% of the analyzed transformants contained the correctly assembled constructs when the number of gRNAs was under 16, and the positive rate decreased to below 10% when the number of gRNAs was 16 or more (Figure 4a). Two transformants were randomly selected from each replicate and ordered. The orientation of the constructs was verified using Sanger sequencing. Overall, we demonstrate through this method that the PARA method is an effective approach for the one-pot assembly of gRNA arrays with up to 16 gRNAs.

In addition to the tRNA-gRNA system, polycistronic transcripts can also be processed post-transcriptionally into individual gRNAs by other RNA-cleaving enzymes, such as the CRISPR-associated RNA endoribonuclease Csy4 [16] and ribozymes (RBs) [17]. Recently, multiplexed CRISPR/Cas9 genome editing has been successfully applied in yeast [18], human cells [19] and plants [5]. We tested the PARA method for the assembly of gRNA arrays based on Csy4 and RB expression systems, and we compared the cloning efficiency of gRNA arrays containing the same set of eight gRNAs in different gRNA expression systems based on tRNA, Csy4 and RB (Figure 4b,c ). All component DNA fragments were generated by either PCR amplification of the predesigned template vector type I or annealing oligonucleotides. High-efficiency cloning was achieved in the Csy4 system (80.0%), tRNA system (73.4%) and RB system (63.0%) (Figure 4c). Recently, it was reported that multiplexed CRISPR/Cas12a was able to target multiple sites with high biallelic editing efficiency in rice using the processing system of the hammerhead (HH) and hepatitis delta virus (HDV) RBs (Figure 4b) [9]. However, the assembly of such a sophisticated construct is difficult and time-consuming. In this study, we sought to create gRNA arrays containing the same components in a one-step effort using the PARA method. Required oligonucleotides were designed manually using the same strategy as shown in Figure 3b. Component fragments were generated through PCR amplification using a predesigned template vector type II (Figure S1b and Table S2). In the 8-gRNA assembly, approximately 36.4% of the analyzed transformants contained the correctly assembled construct (Figure 4c ). Other than PCR amplicons, the HH-HDV-RB array with eight gRNAs was also assembled successfully using synthesized DNA fragments (Figures S3e and S5). Altogether, the PARA method is a potent and robust approach for assembling gRNA arrays with different expression systems.

Based on the literature, multiple tRNA systems with organism-specific tRNA sequences have been used in plants [5,8], yeast [3] and *Drosophila* [4]. The Csy4 system has been used in plants [5], yeast [18] and human cells [19]. The RB and HH-HDV-RB systems have also been used in plants [5,9]. To simplify vector design and construction, we developed a dedicated web tool, PARAweb, which allows users to accurately design and simulate complex cloning procedures that involve numerous gRNAs. PARAweb can be freely accessed at https://fair.ornl.gov/BioDesign/para/para/ (accessed on 17 June 2022). The PARAweb tool is suitable for the design of all of the described gRNA array expression systems (i.e., tRNA, Csy4 and RB for Cas9, as well as HH-HDV-RB for Cas12a) (Figure 4d). Moreover, this web-based gRNA array tool is useful for the application of multiplexed CRISPR knockout, base editing, CRISPRa and CRISPRi in a wide variety of organisms, including animals, plants and microbes. With given input gRNA sequences, PARAweb can generate PCR primers, component fragments and linear assembled gRNA array sequences (Figure 4e). When a valid destination vector sequence is given, PARAweb can also generate the assembled vector sequences containing the gRNA array (Figure 4e ). Notably, the ligation frequency for each OH pair in assembly reactions with BsaI-HFv2 and T4 DNA ligase [7] is utilized as a basic rule to select high-fidelity OH sets in PARAweb. Eight poplar gRNAs used previously were selected to test PARAweb, generating PCR primers, component fragments, linear assembled gRNA array sequences and assembled vector sequences (Figure S6 and Table S3). These component fragments were successfully PCR-amplified with the primers and ligated through linear ligation or cloned into a modified pKSE401 vector in SnapGene (Figure S7). Following the procedures described in Figure 3, we detected 55.6% positive colonies in three biological replicates (Figures S3f and S8).

## 4. Discussion

**Figure 5:**
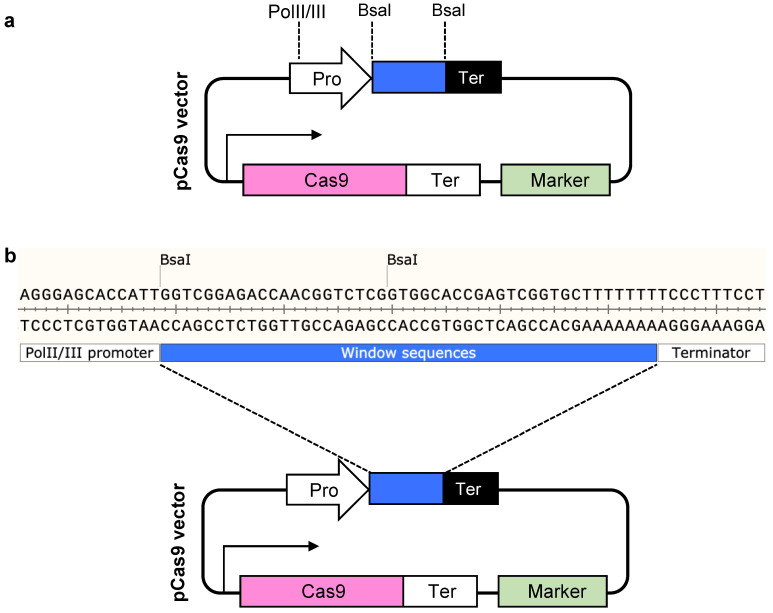
Valid Cas9 vector that is compatible with the PARA method. (**a**) The valid vector must and can only contain two BsaI recognition GGTCTC(1/5)^ sites between the PolII/III promoter and the corresponding terminator. (**b**) A predefined window sequence (GGTCGGAGACCAACGGTCTCGGTGGCACCGAGTCGGTGCTTTTTTT) was used to code PARAweb. To export an assembled vector sequence using PARAweb, the given vector should contain the indicated window sequence.

In summary, we developed the PARA method for fast, efficient, one-step construction of diverse gRNA arrays to facilitate multiplexed genome editing and gene regulation in a wide variety of organisms. Using a minimal set of parts, the approach expands the number of gRNAs that can be assembled into the gRNA arrays in one-pot reaction. Furthermore, we presented the PARAweb tool for optimal design of high-fidelity OHs from a list of gRNA sequences. The PARAweb tool displays ready-to-use primers for the PCR amplification of component fragments along with the simulation of cloning steps. Four gRNA array systems (i.e., tRNA/Csy4/ribozyme/HH-HDV-RB) widely used for CRISPR-Cas-based multiplexed genome editing were programmed into PARAweb for a range of organisms (Table S4). The component DNA parts generated from PARAweb can be directly synthesized via commercial DNA fragment synthesis (e.g., IDT or Twist Bioscience) without requiring additional sequence optimization, which enables the high-throughput gRNA array library assembly. Notably, PARA method can be incorporated with any other vectors that contain two BsaI recognition sites between the promoter and the terminator of a gRNA-array transcription unit (Figure 5a). In addition, a valid destination (CRISPR) vector, in which there is a predefined window sequence flanked by the promoter and the terminator of a gRNA-array transcription unit, is a prerequisite for vector ligation via PARAweb (Figure 5b). As a flexible, universal and all-inclusive methodology for joining gRNA arrays, PARA is dedicated to accelerating the development and application of multiplexed CRISPR in agriculture, medicine and bioenergy in the future.

## Data Availability

The templated vectors and destination vector will be available at Addgene.

## Supplementary Materials

The following are available online at https://www.mdpi.com/article/10.3390/cells11162467/s1, Figure S1: Structure of template vectors for PCR amplification of component DNA fragments; Figure S2: PCR products for the assembly of 8 gRNAs in a plant tRNA system; Figure S3: Colony plates for the assembly of different numbers of gRNAs in different expression systems; Figure S4: Colony PCR for the screening of transformants for the plant tRNA expression system; Figure S5: Colony PCR for the screening of transformants for the plant HH-HDV-RB expression system; Figure S6: PARAweb-assisted assembly of the gRNA array using the plant tRNA expression system; Figure S7: Golden Gate assembly of the plant tRNA-gRNA array using PARA-exported oligos in SnapGene; Figure S8: Colony PCR for the screening of transformants for the plant tRNA expression system; Table S1: Primers and gBlocks used in this study; Table S2: Template vectors type I and II; Table S3: Inputs and outputs of 8-gRNA assembly in PARAweb; Table S4: The parameters used in PARAweb tool.
