# Supplementary material for: PARA: A New Platform for the Rapid Assembly of gRNA Arrays for Multiplexed CRISPR Technologies
Source: Cells. 2022 Aug 9;11(16):2467. doi: 10.3390/cells11162467 (PMC9406951; doi:10.3390/cells11162467)
Supplement: Supplementary file 1 [file cells-11-02467-s001.zip › cells-1841835 Figures S1-S8.pdf]

Supplemental Materials

Supplemental Figures

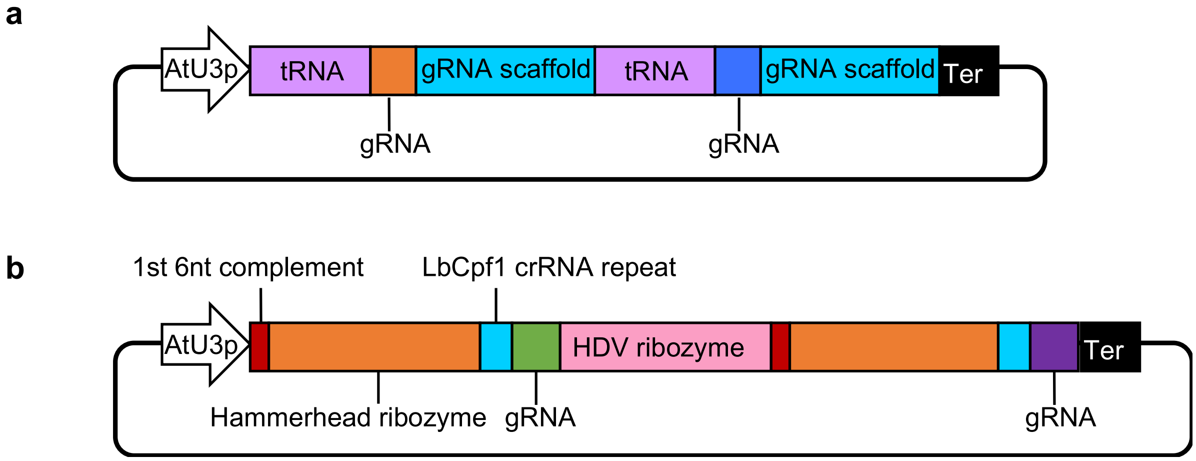

Figure S1. Structure of template vectors for PCR amplification of component DNA fragments. (a) Template vector type I. **(b)** Template vector type II.

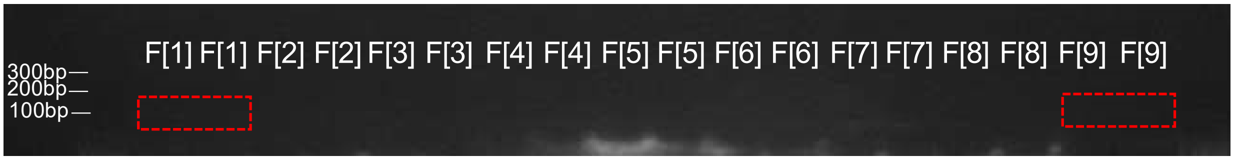

Figure S2. PCR products for the assembly of 8 gRNAs in a plant tRNA system. [n+1] fragments are required for assembly of [n] gRNAs. Two PCR bands were generated for first fragment F[1] and last fragment F[n+1], and red circled bands are the expected fragments.

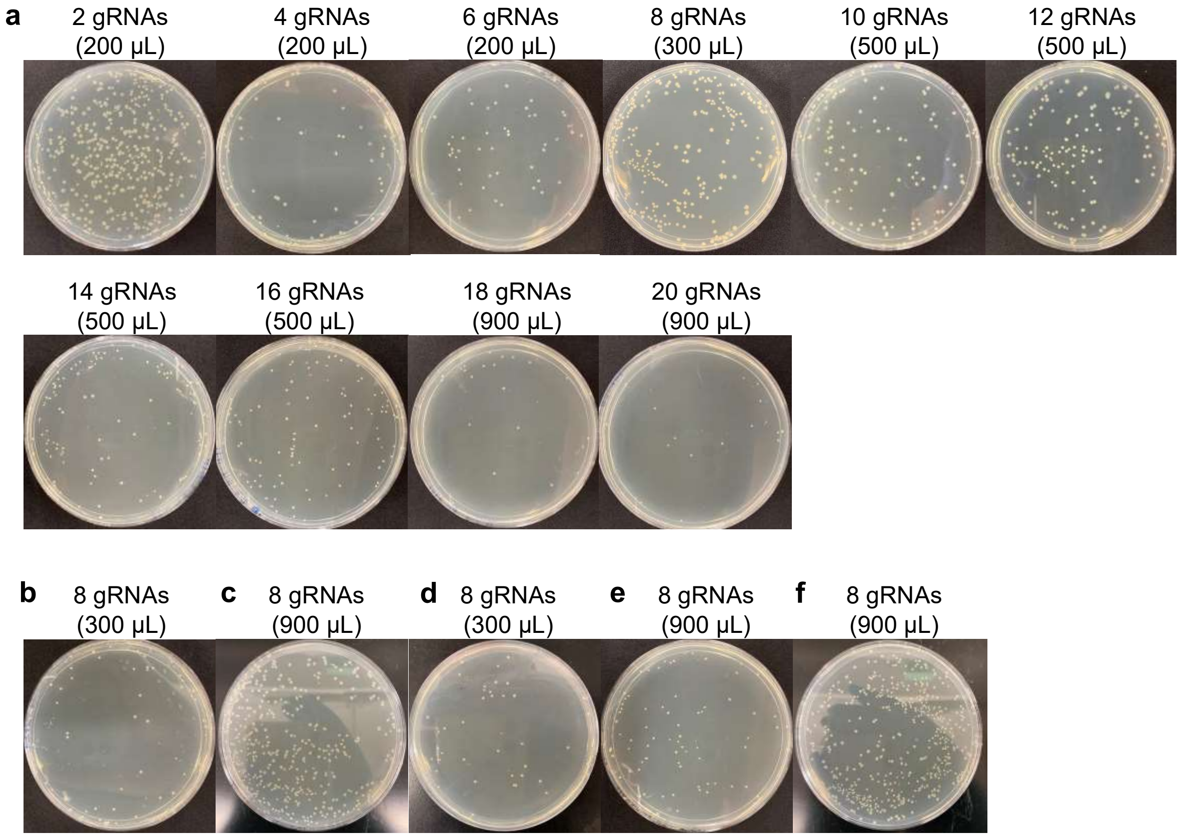

Figure S3. Colony plates for the assembly of different numbers of gRNAs in different expression systems.

(a) Colony plates for the assembly of different numbers of gRNAs in the plant tRNA expression system using PCR products. Loading volume varies in different plates. (**b)** Colony plates for the 8-gRNA assembly in the RB expression system using PCR products. Loading volume = 300 µL. (**c)** Colony plates for the 8-gRNA assembly in the Csy4 expression system using PCR products. Loading volume = 900 µL. (**d)** Colony plates for the 8-gRNA assembly in the HDV-HH-RB expression system using PCR products. Loading volume = 300 µL. (**e)** Colony plates for the 8-gRNA assembly in the HDV-HH-RB expression system using gBlocks. Loading volume = 900 µL. (**f)** Colony plates for the 8-gRNA assembly in the plant tRNA expression system using PARAweb-based PCR products. Loading volume = 900 µL. The total volume of the outgrowth medium is usually 900 µL.

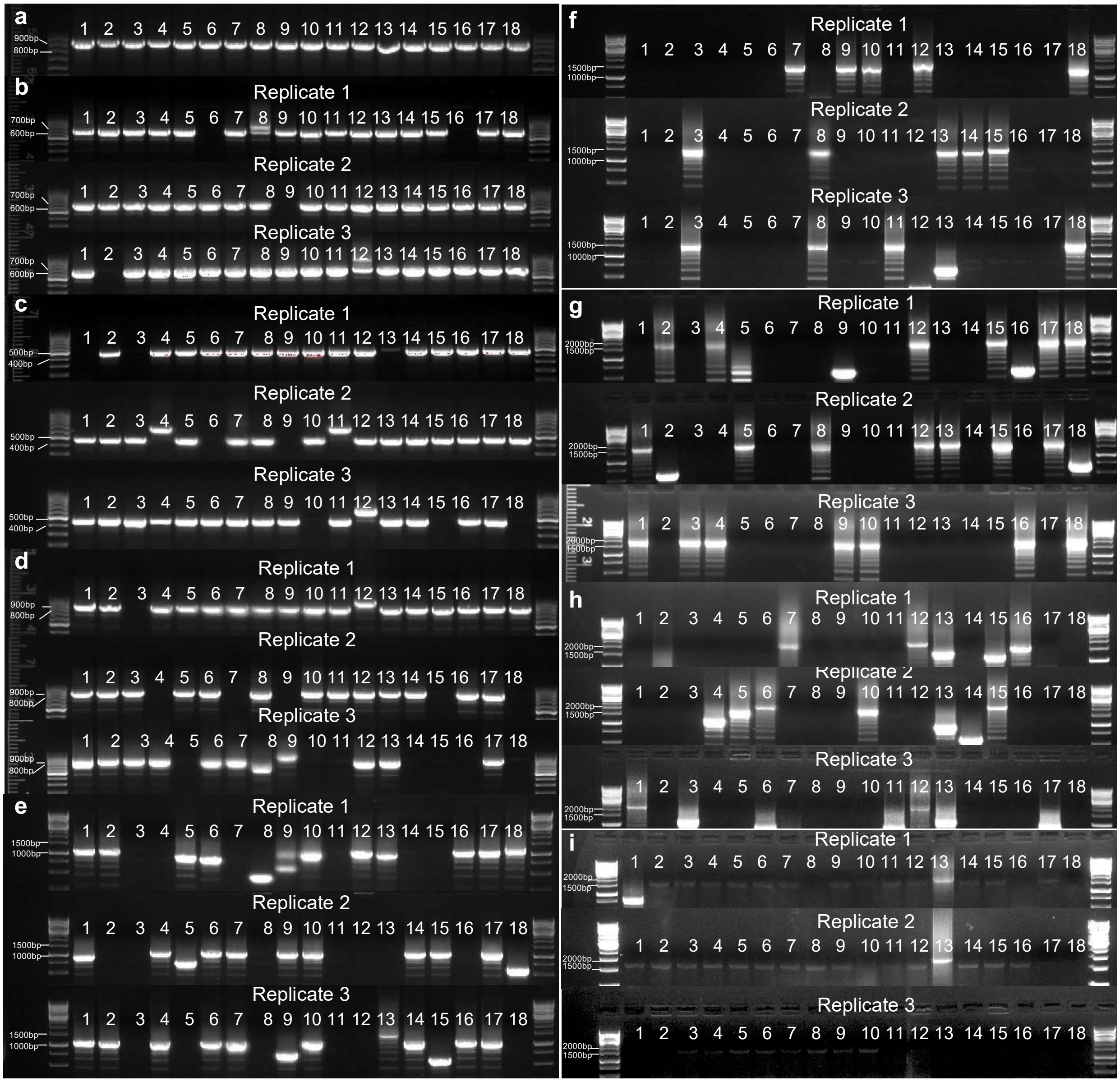

Figure S4. Colony PCR for the screening of transformants for the plant tRNA expression system. (a) Colony PCR of the 2-gRNA assembly. (**b)** Colony PCR of the 4-gRNA assembly. (**c)** Colony PCR of the 6-gRNA assembly. (**d)** Colony PCR of the 8-gRNA assembly. (**e)** Colony PCR of the 10-gRNA assembly. (**f)** Colony PCR of the 12-gRNA assembly. (**g)** Colony PCR of the 14-gRNA assembly. (**h)** Colony PCR of the 16-gRNA assembly. (**i)** Colony PCR of the 18-gRNA assembly.

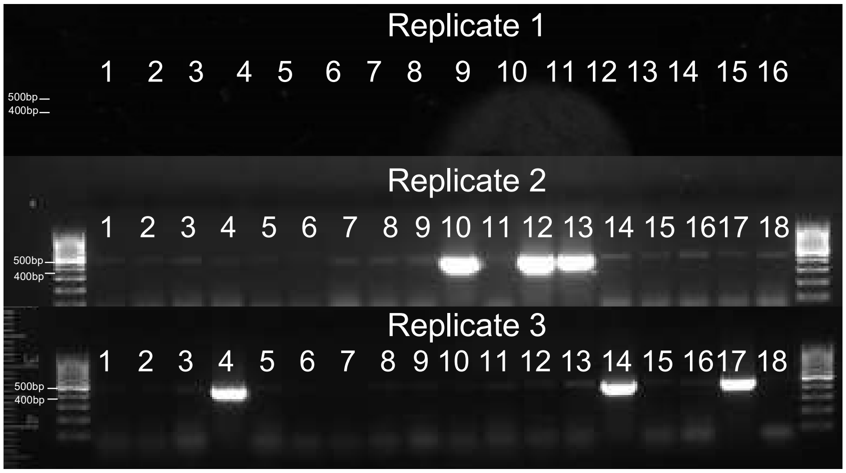

Figure S5. Colony PCR for the screening of transformants for the plant HH-HDV-RB expression system. Colony PCR of the 8-gRNA assembly using synthesized DNA fragments.

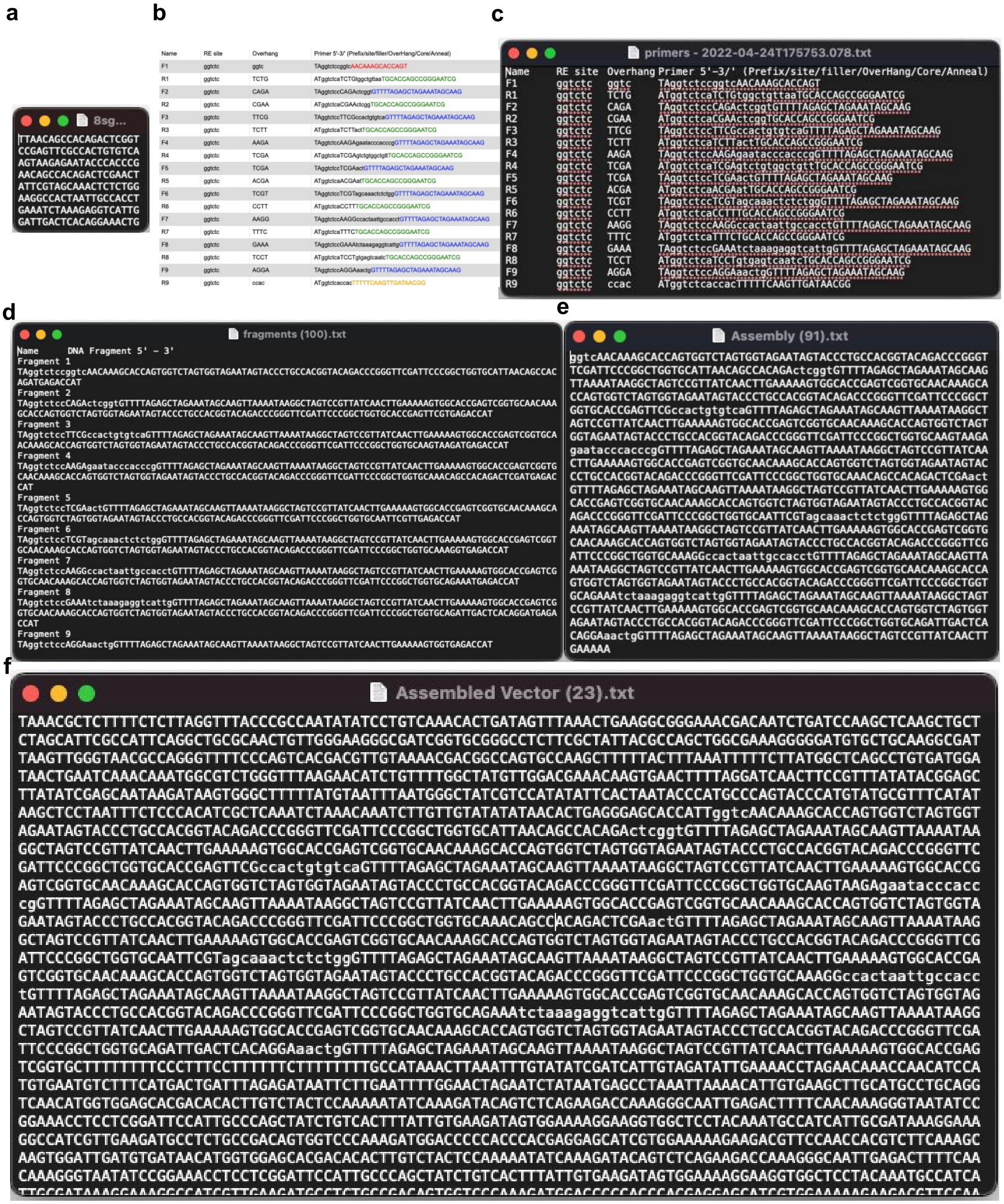

Figure S6. PARAweb-assisted assembly of the gRNA array using the plant tRNA expression system.

(a) The 8-gRNA sequence derived from poplar. **(b)** PARA-generated oligos sequence for PCR-amplified component fragments. **(c)** Downloaded oligos sequence. **(d)** Downloaded component fragments. **(e)** Lineated ligated gRNA array. **(f)** The assembled vector sequence with a given pKSE401 vector.

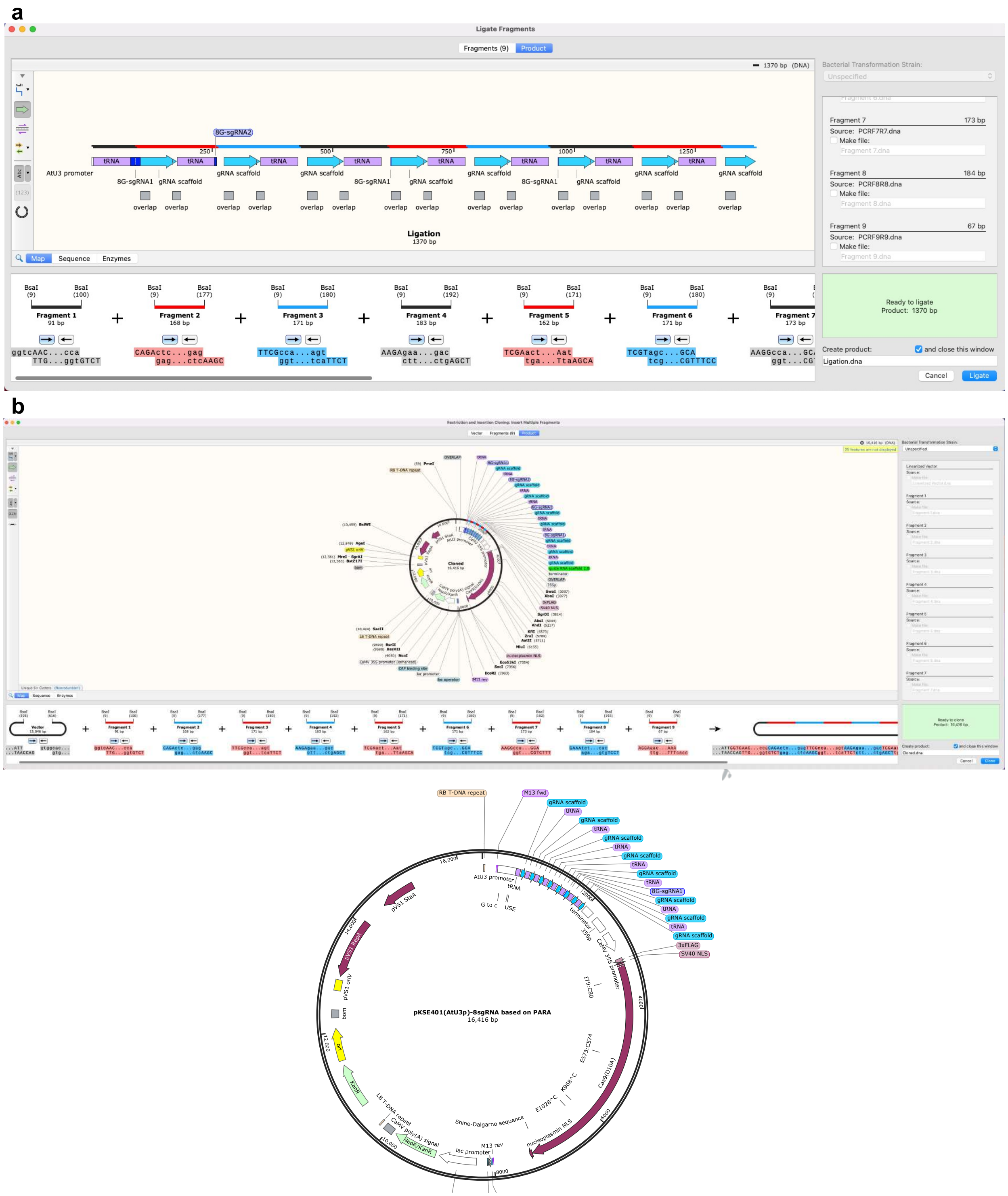

Figure S7. Golden Gate assembly of the plant tRNA-gRNA array using PARA-exported oligos in SnapGene.
(a) Linear ligation. **(b)** Vector cloning.

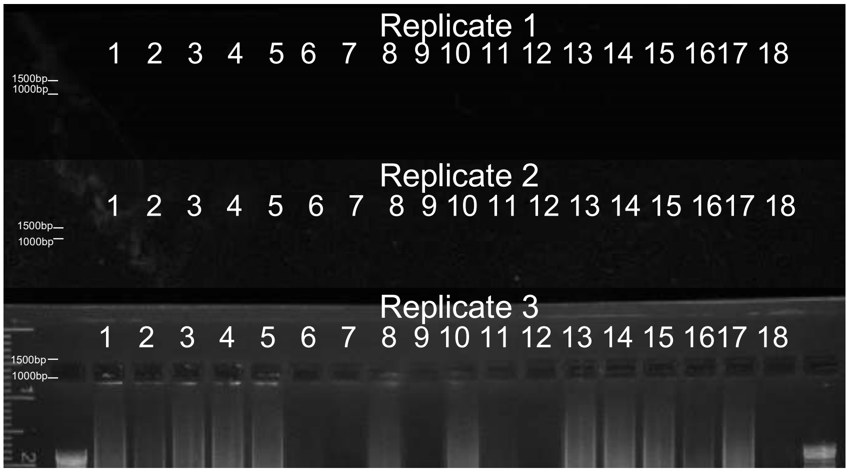

Figure S8. Colony PCR for the screening of transformants for the plant tRNA expression system.

Colony PCR of the 8-gRNA assembly using PARA-designed PCR products.
